# ERAS-guided matrix nursing pathway targeting risk factors in spine surgery: development and prospective evaluation

**DOI:** 10.3389/fmed.2025.1723848

**Published:** 2026-01-08

**Authors:** Yanlan Ma, Jin Zhao, Jing Peng

**Affiliations:** Department of Orthopedics, Chengdu Shangjin Nanfu Hospital (Shangjin Hospital of West China Hospital), Sichuan University, Chengdu, China

**Keywords:** enhanced recovery after surgery, influencing factors, nursing intervention pathway, postoperative complications, spine surgery

## Abstract

**Objective:**

To identify independent risk factors for postoperative infectious complications after spinal surgery and to develop and prospectively evaluate an enhanced recovery after surgery (ERAS)-guided, matrix-structured nursing pathway targeting these risks.

**Methods:**

This mixed-methods study included a retrospective analysis of 220 patients who underwent elective spinal surgery between January 2022 and December 2024. Univariate and multivariable logistic regression analyses were performed to identify independent factors associated with postoperative infectious complications, and predictive performance was assessed using receiver operating characteristic (ROC) analysis. Based on ERAS principles and the identified risk factors, a matrix-structured nursing intervention pathway was developed through multidisciplinary expert consensus. The pathway was then implemented in a prospective cohort of 50 patients (January–June 2025) and compared with 50 matched controls receiving conventional care from the retrospective cohort.

**Results:**

Advanced age, longer operative duration, and greater intraoperative blood loss were independent risk factors, whereas prophylactic antibiotic use was protective (all *p* < 0.05). The combined model demonstrated excellent discrimination (AUC = 0.940). Compared with controls, the intervention group had a lower postoperative complication rate (8% vs. 24%, *p* = 0.029) and shorter operative duration and postoperative hospital stay, with reduced intraoperative blood loss (all *p* < 0.05).

**Conclusion:**

Advanced age, prolonged operative duration, and increased intraoperative blood loss were independently associated with postoperative infectious complications, while prophylactic antibiotic use was protective. The ERAS-guided, matrix-structured nursing pathway was feasible and was associated with improved perioperative outcomes, supporting its potential value for optimizing perioperative care in spinal surgery.

## 1 Introduction

Spinal disorders represent one of the most prevalent clinical issues worldwide, commonly including cervical spondylosis, lumbar spinal stenosis, intervertebral disc herniation, and degenerative spinal diseases (1–3). With the accelerating aging of the global population and changes in lifestyle, the incidence of spinal disorders has been increasing annually, significantly impairing patients’ quality of life and social functioning (4, 5). Surgery serves as a critical intervention for spinal disorders after conservative management failed, playing a central role in improving neurological function, alleviating pain, and enhancing overall quality of life (6). However, due to its invasive nature, technical complexity, and prolonged recovery period, spinal surgery is frequently accompanied by a high incidence of complications. Literature reports indicate that postoperative complication rates following spinal surgery range from 10 to 30%, with infections, hemorrhage, pulmonary complications, and urinary tract infections being the most frequently observed (7–9). In addition, some patients may experience persistent or recurrent spinal pain after surgery; the term persistent spinal pain syndrome (PSPS), particularly PSPS type 2, has been proposed to replace the older term “failed back surgery syndrome” (FBSS) (10). These complications not only extend hospitalization and rehabilitation duration but also increase healthcare resource utilization and patient financial burden, potentially leading to functional impairment or the need for reoperation. Therefore, the effective identification and prevention of postoperative complications in spinal surgery represent an urgent challenge in the fields of surgery and nursing.

In recent years, the introduction and promotion of the enhanced recovery after surgery (ERAS) concept have provided new perspectives for perioperative management in surgical patients (11, 12). ERAS emphasizes the implementation of a series of evidence-based optimized measures aimed at minimizing surgical stress responses, shortening recovery time, and reducing complication risks, without compromising therapeutic efficacy. Extensive research has demonstrated the clinical benefits of ERAS in colorectal surgery, orthopedic joint arthroplasty, and certain neurosurgical procedures (13–15). However, due to the complexity of spinal pathologies, significant inter-patient variability, and diversity in surgical techniques, the development of nursing intervention pathways under the ERAS framework for spinal surgery remains in its exploratory stages. Although some studies have attempted to integrate ERAS measures into the perioperative care of spinal patients, most have focused on isolated aspects, such as preoperative education or early postoperative mobilization, lacking systematic identification of high-risk factors and the construction of comprehensive, risk-based nursing pathways (16, 17). Therefore, beyond demonstrating the general benefits of ERAS, there remains a practical need for an implementation framework that is operable at the bedside and tailored to heterogeneous risk profiles in spine surgery. Compared with conventional ERAS bundles that are often implemented as a largely uniform checklist, a matrix-structured pathway explicitly maps patient- and surgery-specific risk factors to phase-specific nursing actions across the perioperative timeline, with predefined triggers, responsibilities, and operational criteria. This structure facilitates risk-stratified prioritization and standardized workflow execution, thereby enhancing the operability and nursing sensitivity of ERAS implementation in spine surgery. Accordingly, nurse-led, matrix-structured perioperative interventions that systematically target key complication-related risk factors within an ERAS framework remain underexplored in spine surgery. We hypothesized that an ERAS-guided matrix nursing pathway targeting these risk factors would reduce postoperative complications and shorten postoperative length of stay compared with conventional care.

Through retrospective cohort analysis, this study identified independent risk factors for postoperative complications in spinal surgery, including advanced age, prolonged operative duration, and increased intraoperative blood loss, while the rational use of prophylactic antibiotics was confirmed as a protective factor. Building on these findings, the research team innovatively developed a matrix-based nursing intervention pathway, centered on the ERAS concept and integrated with key risk factors. This pathway horizontally encompasses influencing factors and vertically spans the entire perioperative process (preoperative, intraoperative, and postoperative phases), emphasizing individualized, structured, and evidence-based nursing measures. The objective is to reduce the incidence of complications, shorten operative duration and hospital stays, and facilitate rapid recovery through targeted management.

Thus, the significance of this study lies not only in identifying modifiable risk factors for complications in spinal surgery but also in integrating these factors with practical interventions via an ERAS-based nursing pathway. This approach provides a systematic and actionable reference for perioperative nursing management in spinal surgery patients, offering substantial clinical value and broad applicability for advancing the implementation of ERAS in spinal surgery, enhancing nursing quality, and improving patient outcomes.

## 2 Materials and methods

### 2.1 Study design

This study employed a mixed-methods, two-phase design consisting of a retrospective risk-factor identification phase followed by a prospective, before-after evaluation phase. In addition, a qualitative pathway-development component was conducted, including evidence synthesis and an expert-panel consensus process to construct and refine the ERAS-guided matrix-structured nursing pathway. First, a retrospective cohort was established using historical data from patients who underwent elective spinal surgery in our department between January 2022 and December 2024 to identify independent factors associated with postoperative complications. Based on these risk factors and ERAS principles, we developed an ERAS-guided, matrix-structured nursing intervention pathway that standardized perioperative assessment and targeted risk mitigation.

**Figure 1:**
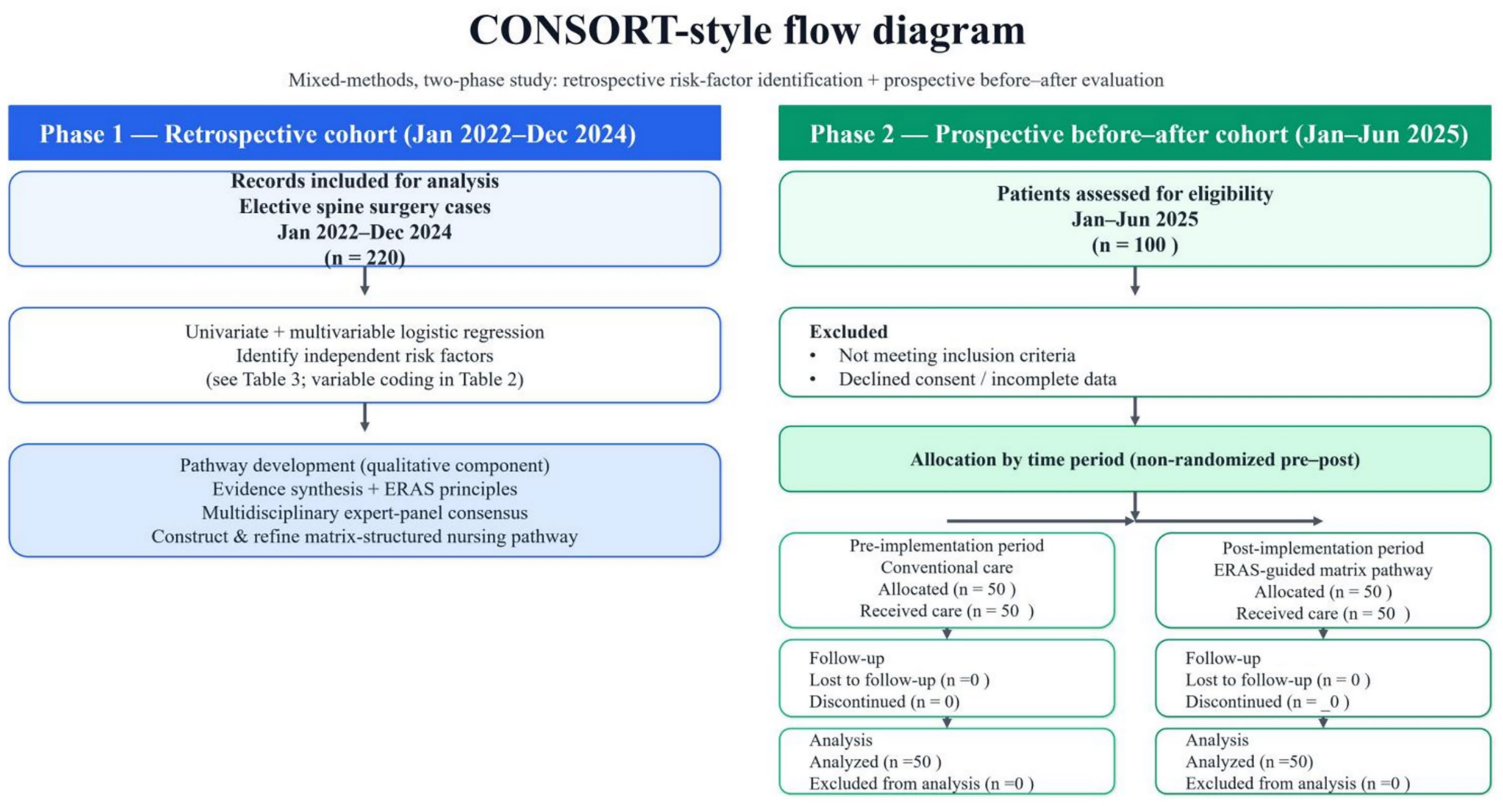
CONSORT-style flow diagram.

Subsequently, the pathway was implemented and evaluated in a prospective cohort between January 2025 and June 2025 using a non-randomized, time-period (pre-post) controlled design. Patients treated during the pre-implementation period received conventional perioperative care, whereas those treated during the post-implementation period received the ERAS-guided matrix nursing pathway. To enhance transparency, patient inclusion/exclusion and cohort formation are summarized in the CONSORT-style flow diagram (Figure 1). Given the non-randomized before–after nature of the study, we minimized potential confounding by applying consistent eligibility criteria and perioperative management standards across periods within the same department, and we further accounted for baseline differences using prespecified multivariable-adjusted analyses (covariates including demographic characteristics, comorbidities, and key surgical factors). The study protocol was approved by the Ethics Committee of our hospital. Written informed consent was obtained from all participants enrolled in the prospective phase.

### 2.2 Study population and sample size

#### 2.2.1 Participant recruitment

Patients who underwent elective spinal surgery in our department during two distinct periods were selected: a retrospective cohort (January 2022–December 2024) and a prospective cohort (January 2025–June 2025).

#### 2.2.2 Inclusion and exclusion criteria

##### 2.2.2.1 Inclusion criteria

1. Age ≥18 years.
2. Diagnosed with a spinal disorder (18) meeting surgical indications and scheduled for open or minimally invasive spinal surgery.
3. Classified as American Society of Anesthesiologists (ASA) physical status (19) I–III.
4. Conscious, without communication barriers, and able to cooperate with surveys and follow-up assessments.
5. Provided informed consent and voluntarily agreed to participate in the study.

##### 2.2.2.2 Exclusion criteria

1. Patients undergoing emergency surgery, revision surgery, or those with associated major trauma.
2. Comorbid severe cardiac, pulmonary, hepatic, or renal insufficiency, or coagulation disorders.
3. History of malignant tumors, immune system diseases, or psychiatric disorders.
4. Pregnant or lactating women.
5. Patients with incomplete clinical data or loss to follow-up.

#### 2.2.3 Sample size calculation

For the retrospective cohort, a target sample size of 220 patients was planned, based on the annual surgical volume in previous years. This number satisfies the requirement for a sample size 5 to 10 times the number of independent variables (influencing factors) for the planned multivariate logistic regression analysis (20), thereby ensuring model stability. For the prospective cohort, sample size estimation was based on a pilot study indicating that the incidence of postoperative complications could decrease from approximately 25% in the conventional care group to 8% after implementation of the ERAS-based nursing pathway. Under a two-sided test with a significance level of *α* = 0.05 and statistical power of 1−*β* = 0.80, the required sample size for comparing two independent proportions was calculated to be 45 participants per group. Considering an anticipated 10% loss to follow-up or incomplete data, the final planned enrollment was set at 50 patients per group (a total of 100), which was deemed sufficient for preliminary comparative effectiveness evaluation. Given that the prospective cohort focused on pathway implementation and preliminary assessment, a convenience sampling method was adopted. Accordingly, 50 patients were consecutively enrolled into the intervention group, while another 50 matched controls were selected from the retrospective cohort to allow for an initial comparison of clinical outcomes and pathway effectiveness. The intervention-phase sample size was calculated to detect a between-group difference in the primary outcome (postoperative infectious complication rate/overall complication incidence). Secondary outcomes were not individually powered; therefore, analyses of secondary endpoints should be interpreted as exploratory.

### 2.3 Research methods

#### 2.3.1 Data collection and statistical analysis

Data were extracted from the electronic medical record system and encompassed: demographic and baseline characteristics [age, sex, body mass index (BMI), smoking history, alcohol history]; comorbidities (e.g., diabetes, hypertension); nutritional and functional status (preoperative hypoalbuminemia, anemia); and surgery-related variables [surgical site, operative levels, surgical approach (open/minimally invasive), operative duration, intraoperative blood loss, intraoperative transfusion volume]. Outcome variables included infectious complications occurring within 30 days postoperatively, such as surgical site infection, pulmonary infection, and urinary tract infection. Infectious complications were selected *a priori* as the primary postoperative event endpoint because they are among the most frequent and nursing-sensitive complications after spine surgery and can be directly targeted by perioperative risk-mitigation measures. Other postoperative events (e.g., thromboembolism, neurological deficit) were not included in the primary complication endpoint due to their lower incidence and differing etiologies, which would require larger samples for adequately powered analyses.

#### 2.3.2 Intervention application and evaluation

Building upon the independent risk factors identified via multivariate logistic regression analysis, a matrix-based nursing intervention pathway was constructed, guided by the core principles of ERAS (21). Pathway construction followed a structured procedure: (1) mapping identified risk factors and core ERAS elements to perioperative phases; (2) drafting risk-triggered nursing actions with explicit operational criteria (e.g., thresholds, timing, responsibilities); and (3) iterative refinement through multidisciplinary expert-panel review to ensure content validity, feasibility, and consistency with routine clinical workflow. This pathway was structured along a horizontal axis representing key influencing factors and a vertical axis spanning the perioperative phases (preoperative, intraoperative, postoperative), aiming to deliver targeted interventions through structured, evidence-based measures. The integrated nursing pathway was implemented in the prospective cohort (intervention group, *n* = 50), whose outcomes were compared with those of baseline-matched patients from the retrospective cohort (control group, *n* = 50). Primary evaluation metrics included pathway feasibility (e.g., protocol adherence rate) and preliminary effectiveness (incidence of complications, operative duration, intraoperative blood loss, and time to first ambulation).

#### 2.3.3 Statistical analysis

All analyses were performed using SPSS version 26.0 (IBM Corp., Armonk, NY, United States). Continuous variables were expressed as mean ± standard deviation (x ± s) or median (interquartile range) [M (IQR)], and compared using the *t*-test or Mann–Whitney U test, as appropriate. Categorical variables were summarized as number (percentage) [*n* (%)] and analyzed using the χ^2^ test or Fisher’s exact test. Univariate and multivariate logistic regression analyses were employed to identify independent factors associated with postoperative complications. Variables entered into the multivariable model were selected based on both clinical relevance (*a priori* potential confounders) and univariate screening (*p* < 0.05), rather than relying solely on statistical significance. A forced-entry (Enter) approach was used for the prespecified covariates, and multicollinearity was assessed using variance inflation factors (VIF), with VIF < 5 indicating acceptable collinearity. Model performance was evaluated by calibration using the Hosmer-Lemeshow goodness-of-fit test. Receiver operating characteristic (ROC) curve analysis was conducted to evaluate the predictive value of significant factors for postoperative complications. A *p*-value < 0.05 was considered statistically significant.

## 3 Results

### 3.1 Univariate analysis of postoperative complications following spinal surgery

Univariate analysis identified several factors significantly associated with the occurrence of postoperative complications after spinal surgery (*p* < 0.05), including age, history of diabetes, preoperative hypoalbuminemia, operative duration, intraoperative blood loss, and the use of prophylactic antibiotics. The detailed results are presented in Table 1.

**Table 1:** Univariate analysis of factors associated with postoperative complications after spinal surgery.

Variable	Total (*n* = 220)	Non-complication group (*n* = 168)	Complication group (*n* = 52)	*t/χ^2^*	*P*
Age (years)	56.83 ± 5.38	55.65 ± 5.34	60.63 ± 3.41	−6.334	<0.001
Sex, *n* (%)				0.021	0.884
Male	125 (56.82)	95 (56.55)	30 (57.69)		
Female	95 (43.18)	73 (43.45)	22 (42.31)		
BMI (kg/m^2^)	24.97 ± 1.22	24.92 ± 1.19	25.13 ± 1.31	−1.079	0.282
Smoking history, *n* (%)	74 (33.63)	54 (32.14)	20 (38.46)	0.710	0.399
Alcohol history, *n* (%)	97 (44.09)	75 (44.64)	23 (44.23)	0.003	0.958
Diabetes, *n* (%)	51 (23.18)	32 (19.05)	19 (36.54)	6.822	0.009
Hypertension, *n* (%)	87 (39.55)	67 (39.88)	20 (38.46)	0.033	0.855
Hypoalbuminemia, *n* (%)	57 (25.91)	36 (21.43)	21 (40.38)	7.433	0.006
Anemia, *n* (%)	41 (18.64)	30 (17.86)	11 (21.15)	0.285	0.594
Surgical site, *n* (%)				0.981	0.612
Thoracic	30 (13.64)	25 (14.88)	5 (9.62)		
Cervical	26 (11.82)	20 (11.90)	6 (11.54)		
Lumbar	164 (74.55)	123 (73.21)	41 (78.85)		
Operative duration (min)	156.45 ± 25.45	158.49 ± 22.27	182.19 ± 16.49	−10.085	<0.001
Intraoperative blood loss (ml)	320.99 ± 77.27	310.00 ± 76.76	356.52 ± 68.26	−3.916	<0.001
Operative levels, *n* (%)				0.001	0.969
Single-level	157 (71.36)	120 (71.43)	37 (71.15)		
Multi-level	63 (28.64)	48 (28.57)	15 (28.85)		
Surgical approach, *n* (%)				0.404	0.525
Open surgery	172 (78.18)	133 (79.17)	39 (75.00)		
Minimally invasive surgery	48 (21.82)	35 (20.83)	13 (25.00)		
Prophylactic antibiotics, *n* (%)	169 (76.82)	140 (83.33)	29 (55.77)	16.942	<0.001

BMI, Body mass index.

### 3.2 Logistic regression analysis of factors influencing postoperative complications after spinal surgery

A multivariable logistic regression model was constructed with covariates chosen by clinical *a priori* confounders and univariate screening (*p* < 0.05). Key confounders (ASA class, nutritional indicators such as hypoalbuminemia/anemia, and surgery-related characteristics including approach/levels/site) were forced into the model using the Enter method, and multicollinearity was acceptable across predictors (all VIF < 5). Using the occurrence of postoperative infectious complications as the dependent variable (coded as 0 for no occurrence, 1 for occurrence), variables showing significant differences in the univariate analysis were included as independent variables in a multivariate logistic regression model (assignment methods detailed in Table 2). The analysis identified advanced age (OR = 1.254 per 1-year increase, 95% CI: 1.117–1.408, *p* < 0.001), prolonged operative duration (OR = 1.087 per 1 min increase, 95% CI: 1.054–1.120, *p* < 0.001), and greater intraoperative blood loss (OR = 1.009 per 1-mL increase, 95% CI: 1.002–1.015, *p* = 0.013) as independent risk factors for postoperative complications. In contrast, the administration of prophylactic antibiotics (OR = 0.164, 95% CI: 0.053–0.511, *p* = 0.002) were independently associated with postoperative infectious complications. For clinical interpretability, the operative-time effect corresponds to an OR of approximately 12.08 per 30 min increase (derived from OR_1min^30). Model calibration was acceptable (Hosmer-Lemeshow goodness-of-fit test: χ^2^ = 11.910, df = 8, *p* = 0.155). The complete regression results are presented in Table 3.

**Table 2:** Variable assignment methods for logistic regression analysis.

Variable	Assignment
Age	Continuous variable
Operative duration	Continuous variable
Intraoperative blood loss	Continuous variable
Prophylactic antibiotics	No = 0, Yes = 1
Diabetes	No = 0, Yes = 1
Hypoalbuminemia	No = 0, Yes = 1

**Table 3:** Multivariate logistic regression analysis of factors associated with postoperative complications after spinal surgery.

Variable	*B*	SE	Wald	*P*	OR	95% CI	
						Lower	Upper
Age	0.227	0.059	14.685	<0.001	1.254	1.117	1.408
Operative duration	0.083	0.016	28.695	<0.001	1.087	1.054	1.120
Intraoperative blood loss	0.008	0.003	6.177	0.013	1.009	1.002	1.015
Prophylactic antibiotics	−1.808	0.580	9.720	0.002	0.164	0.053	0.511
Diabetes	0.947	0.568	2.784	0.095	2.578	0.848	7.842
Hypoalbuminemia	0.881	0.565	2.425	0.119	2.412	0.796	7.307

### 3.3 Predictive value of independent factors for postoperative complications

**Figure 2:**
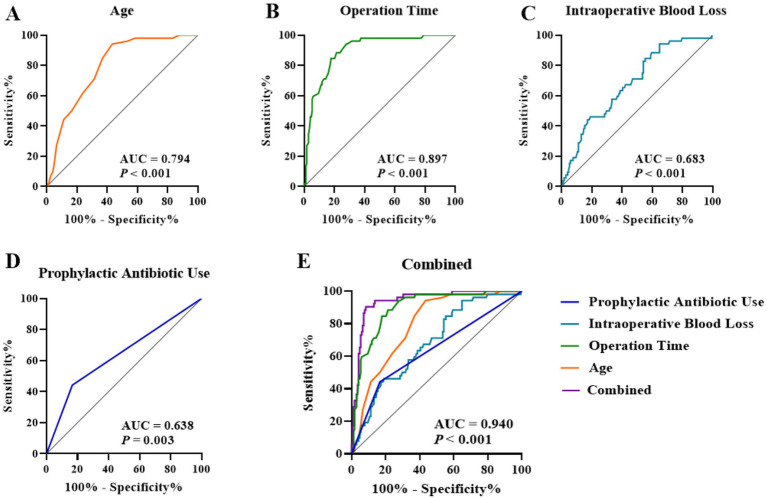
ROC curves of independent factors for predicting postoperative complications: **(A)** Age, **(B)** Operation time, **(C)** Intraoperative blood loss, **(D)** Prophylactic antibiotic use, and **(E)** Combined model.

To evaluate the predictive performance of the identified independent factors, ROC curves were constructed. The results demonstrated the following: Age (Figure 2A) yielded an area under the curve (AUC) of 0.794 (95% CI: 0.732–0.856), with an optimal cutoff value of > 56.50 years, a sensitivity of 94.23%, and a specificity of 56.55%. Operative duration (Figure 2B) achieved an AUC of 0.897 (95% CI: 0.852–0.943), with an optimal cutoff of >165.5 min, corresponding to a sensitivity of 88.46% and a specificity of 78.57%. Intraoperative blood loss (Figure 2C) produced an AUC of 0.683 (95% CI, 0.604–0.761), with an optimal cutoff of > 277.0 mL, a sensitivity of 94.23%, and a specificity of 35.12%. The AUC for prophylactic antibiotic use (Figure 2D) was 0.638 (95% CI, 0.546–0.730). Notably, the combined predictive model incorporating all four factors (Figure 2E) exhibited a substantially improved AUC of 0.940 (95% CI, 0.906–0.975), indicating excellent predictive capability. Internal validation using bootstrap resampling (5,000 repetitions) yielded a similar AUC with a 95% confidence interval of 0.907–0.972. In addition, stratified 10-fold cross-validation demonstrated stable performance (mean AUC = 0.952, SD = 0.045; range 0.882–1.000).

### 3.4 Development of an ERAS-based nursing intervention pathway integrated with independent influencing factors

Guided by the core principles of ERAS and building upon the independent risk factors (advanced age, prolonged operative duration, and high intraoperative blood loss) and protective factor (use of prophylactic antibiotics) identified in the previous multivariate logistic regression analysis, a matrix-based nursing intervention pathway was constructed. This pathway is structured along a horizontal axis representing the key influencing factors and a vertical axis encompassing the preoperative, intraoperative, and postoperative phases, aiming to deliver targeted interventions through structured measures. The pathway was developed and finalized through multidisciplinary discussions involving expert panels from orthopedics, anesthesiology, nutrition, and nursing. Importantly, the matrix format was designed to operationalize ERAS implementation by converting broad ERAS recommendations into risk-triggered, phase-specific nursing tasks with explicit criteria, rather than a uniform bundle applied identically to all patients. The core components of the intervention pathway are detailed in Table 4.

**Table 4:** Perioperative nursing intervention pathway for spinal surgery based on ERAS principles and complication influencing factors.

Perioperative phase	Intervention goal	Specific interventions and implementation criteria
Preoperative	1. Optimize physiological reserve in elderly patients (targeting advanced age)	Comprehensive geriatric assessment: Performed by a specialized nurse within 3 days preoperatively for patients aged ≥ 56 years, covering cognition, nutrition, mobility, and comorbidities. Individualized prehabilitation: Respiratory training: 3 times daily, 10–15 min/session, instructing deep breathing (inhale 4–5 s, exhale 6–7 s) and effective coughing. Lower limb exercise: Ankle pump exercises, straight leg raises; 10–15 repetitions/exercise, 3 sets/day. Education & Psychological support: Clearly explain rehabilitation goals and pain management plan to alleviate anxiety.
	2. Strengthen nutritional foundation (targeting risks, e.g., hypoalbuminemia)	Nutritional screening & support: Complete NRS-2002 screening within 24 h of admission. For patients with ALB <35 g/L, develop an individualized diet plan (daily protein intake 1.2–1.5 g/kg) and recommend oral nutritional supplements; consult nutrition department if necessary.
Intraoperative	3. Minimize surgical trauma and stress (targeting operative duration & blood loss)	Process optimization & team coordination: Instrument nurse participates in surgical plan discussion 1 day prior; prepare minimally invasive instruments to reduce intraoperative delays. Comprehensive temperature protection: Use warming blankets on non-surgical areas; maintain all IV fluids and irrigation fluids at 37 °C using fluid warmers; target core temperature >36 °C. Precise fluid management: Collaborate with anesthesiologist for goal-directed fluid therapy to maintain hemodynamic stability.
	4. Standardize infection prevention (reinforcing protective factor)	Precise antibiotic administration: Strictly adhere to guidelines; complete IV administration within 0.5–1 h before skin incision. Administer an additional dose if operative duration >3 h or blood loss >1,500 mL.
Postoperative	5. Promote rapid functional recovery (comprehensive approach to age/trauma)	Multimodal analgesia: Base regimen: “NSAIDs (e.g., celecoxib 200 mg bid) + acetaminophen.” Assess VAS score every 4 h postoperatively; combine short-acting opioids per physician’s order if VAS ≥ 4. Individualized early mobilization: Within 6 h postop: Independent bed turning, ankle pump exercises. Postop day 1: Sit on bed edge with assistance for ≥ 10 min, twice daily. Postop day 2: Assisted standing and slow walking 5–10 m, 2–3 times daily (implement after strict assessment for elderly patients). Early nutritional support: Clear liquids (water, rice soup) 50 mL at 4 h postop; repeat after 2 h if tolerated. Transition to high-protein semi-liquid diet (e.g., minced meat porridge, fish soup) on postop day 1.

### 3.5 Baseline characteristics

No statistically significant differences were observed in baseline characteristics between the intervention and control groups, including age, sex, BMI, and surgical types (all *p* > 0.05), indicating that the groups were comparable. The detailed comparisons are presented in Table 5.

**Table 5:** Comparison of baseline characteristics between the control and intervention groups.

Variable	Total (*n* = 100)	Control group (*n* = 50)	Intervention group (*n* = 50)	*t/χ^2^*	*P*
Age (years)	56.94 ± 5.49	57.02 ± 5.40	56.86 ± 5.63	0.145	0.885
Sex, *n* (%)				0.649	0.420
Male	56 (56.00)	26 (52.00)	30 (60.00)		
Female	54 (54.00)	24 (48.00)	20 (40.00)		
BMI (kg/m^2^)	24.73 ± 1.42	24.84 ± 1.31	24.62 ± 1.53	0.769	0.444
Smoking history, *n* (%)	38 (38.00)	21 (42.00)	17 (34.00)	0.679	0.410
Alcohol history, *n* (%)	47 (47.00)	25 (50.00)	22 (44.00)	0.361	0.548
Diabetes, *n* (%)	33 (33.00)	18 (36.00)	15 (30.00)	0.407	0.523
Hypertension, *n* (%)	44 (44.00)	20 (40.00)	24 (48.00)	0.649	0.420
Hypoalbuminemia, *n* (%)	31 (31.00)	14 (28.00)	17 (34.00)	0.421	0.517
Anemia, *n* (%)	18 (18.00)	10 (20.00)	8 (16.00)	0.271	0.603
Surgical site, *n* (%)				0.481	0.786
Thoracic	16 (16.00)	9 (18.00)	7 (14.00)		
Cervical	9 (9.00)	5 (10.00)	4 (8.00)		
Lumbar	75 (75.00)	36 (72.00)	39 (78.00)		
Operative levels, *n* (%)				0.735	0.391
Single-level	68 (68.00)	36 (72.00)	32 (64.00)		
Multi-level	32 (32.00)	14 (28.00)	18 (36.00)		
Surgical approach, *n* (%)				0.542	0.461
Open surgery	79 (79.00)	38 (76.00)	41 (82.00)		
Minimally invasive surgery	21 (21.00)	12 (24.00)	9 (18.00)		

### 3.6 Preliminary application outcomes of the nursing intervention pathway

The aforementioned pathway was implemented in 50 patients (intervention group) and compared with 50 patients receiving conventional care (control group). Initial results demonstrated a significantly lower overall postoperative complication rate in the intervention group compared to the control group (8.00% vs. 24.00%, χ^2^ = 4.760, *p* = 0.029). Regarding surgical indicators, the intervention group exhibited significant reductions in operative duration and intraoperative blood loss, alongside a shorter postoperative hospital stay compared to the control group (all *p* < 0.05). The detailed outcomes are presented in Table 6.

**Table 6:** Preliminary outcomes of the nursing intervention pathway implementation.

Group	Complication rate	Operative duration	Intraoperative blood loss	Postoperative hospital stay
Control (*n* = 50)	12 (24.00)	156.66 ± 26.48	337.00 ± 75.18	9.00 (7.00, 10.00)
Intervention (*n* = 50)	4 (8.00)	140.56 ± 10.81	308.54 ± 55.36	6.00 (5.00, 7.00)
*z/t/χ^2^*	4.762	3.980	2.155	−5.225
*P*	0.029	<0.001	0.034	<0.001

## 4 Discussion

This study employed a mixed-method design integrating retrospective and prospective components to first identify independent risk factors for postoperative complications following spinal surgery, and subsequently, guided by the ERAS principles, to develop and prospectively validate a matrix-based nursing intervention pathway. Our findings demonstrated that advanced age, prolonged operative duration, and increased intraoperative blood loss significantly elevate complication risk, whereas the appropriate use of prophylactic antibiotics served as a protective factor. The nursing intervention pathway constructed on this basis effectively reduced complication rates, shortened hospital stays, and contributed to reductions in both intraoperative blood loss and operative time. These results align with previous literature while introducing a novel approach to structuring nursing interventions.

Regarding risk factors, advanced age as an independent risk factor is well-established (22–24). Elderly patients often present with multiple chronic conditions, diminished immune function, and reduced physiological reserve, rendering them more susceptible to infections and functional decline under surgical stress (25). Our study further quantifies the contribution of age to complication risk through regression analysis, underscoring the need for targeted interventions in this demographic. The positive correlation between prolonged operative duration and complications may stem from increased procedural complexity, extended anesthesia time, and prolonged tissue exposure, all of which can elevate infection and bleeding risks (22, 26, 27). Significant intraoperative blood loss can lead to hemodynamic instability, impaired tissue perfusion, and coagulopathy, thereby increasing susceptibility to infections and other complications. Our findings highlight the importance of meticulous blood loss management through refined surgical techniques and goal-directed fluid therapy. Conversely, prophylactic antibiotic administration emerged as a protective factor, consistent with domestic and international guidelines recommending administration within 0.5–1 h before incision to significantly reduce surgical site and pulmonary infection risks (28, 29). Our results reaffirm the importance of protocol adherence, emphasizing strict compliance with timing and indication requirements.

Unlike previous investigations limited to risk factor identification, this study innovatively integrated these factors with ERAS principles to construct a matrix-based intervention pathway. This pathway’s novelty lies in its horizontal-vertical structure linking specific risk factors to targeted interventions across perioperative phases. Preoperative comprehensive geriatric assessment and prehabilitation optimized patients’ physiological readiness through respiratory training, lower limb strengthening, and nutritional optimization, enhancing tolerance to surgical stress (30). Intraoperative measures, including team coordination, temperature protection, and precise fluid management, effectively reduced operative duration and blood loss, while standardized antibiotic administration mitigated infection risks (31). Postoperative multimodal analgesia, early mobilization, and stepwise nutritional support accelerated the recovery of gastrointestinal and systemic functions (32). This structured, phase-specific approach ensures comprehensive coverage and enhances both scientific rigor and operational feasibility. Taken together, these findings support the feasibility of implementing an ERAS-guided, matrix-based nursing pathway in routine spinal surgery care and suggest that the pathway may be associated with improved perioperative management and reduced infectious morbidity.

From a mechanistic perspective, the pathway’s efficacy likely involves multiple interconnected factors. Preoperative optimization of nutritional and functional status enhances physiological reserve and resistance to surgical stress (33). In addition, intraoperative measures preventing hypothermia and excessive blood loss preserve immune function (34). Moreover, postoperative early mobilization and nutritional support mitigate immobilization-related complications while promoting tissue repair and immune recovery (35). These elements form an integrated, closed-loop management system fully aligned with ERAS principles, emphasizing multidimensional optimization throughout the perioperative period to accelerate recovery.

The clinical significance of this study lies in providing a scientifically-grounded nursing model for spinal surgery patients. Pathway implementation facilitates standardized, systematic nursing care, reducing variability and fragmentation while improving overall quality. This model also promotes efficient resource utilization by reducing complications, shortening hospital stays, and lowering readmission rates, thereby alleviating healthcare costs and patient burden. Future implementation should emphasize multidisciplinary collaboration involving surgery, anesthesiology, nursing, and nutrition departments. Furthermore, integration with clinical information systems could enable intelligent reminders and execution monitoring, enhancing compliance and controllability.

Nevertheless, this study has several limitations. First, the prospective component involved a relatively small sample from a single center, potentially limiting generalizability. In addition, the non-randomized, time-period (pre-post) design and the lack of blinding may have introduced selection, performance, and detection bias, and residual confounding cannot be fully excluded. Outcome assessors were not blinded, and potential Hawthorne effects during pathway implementation may also have influenced care processes and outcomes. Future multicenter randomized implementation trials, ideally with blinded outcome assessment, are warranted to confirm the robustness and generalizability of these findings. Second, pathway effectiveness depends on nursing compliance and patient cooperation, which may vary across clinical settings due to differences in staff expertise and individual patient factors. Third, the intervention phase was powered primarily for detecting differences in infectious complication incidence, and the focus on infectious complications may not fully represent the entire spectrum of postoperative morbidity, as other complications such as thrombosis or nerve injury were not comprehensively analyzed. Finally, the short follow-up period necessitates further investigation into the pathway’s long-term impact on functional recovery and sustained outcomes.

Future research should address these aspects through: (1) multicenter, large-sample randomized controlled trials to validate effectiveness and generalizability; (2) incorporation of digital monitoring and artificial intelligence for real-time data acquisition and decision support; (3) expansion of complication profiling to establish comprehensive risk prediction and intervention systems; and (4) development of personalized pathway adaptations for specific populations, such as elderly patients, complex multi-level surgeries, or those with significant comorbidities, to achieve precision nursing management.

## 5 Conclusion

In summary, this study not only identifies key independent risk factors for postoperative complications in spinal surgery but also presents the integration of ERAS principles with risk factor management through a systematic nursing intervention pathway, demonstrating its feasibility and potential effectiveness in a prospective study. Pathway implementation was associated with a lower complication incidence, shorter operative duration and hospital stay, faster recovery, and improved quality of life. Given the study limitations, these findings should be interpreted with caution, and further multicenter studies ideally with randomized design and blinded outcome assessment are warranted to confirm the observed benefits. Despite these limitations, this work provides a novel model and framework for perioperative nursing in spinal surgery with meaningful clinical relevance and potential for broader implementation.

## Data Availability

The original contributions presented in the study are included in the article/supplementary material, further inquiries can be directed to the corresponding author.
